# Modelling the Aggregation Process of Cellular Slime Mold by the Chemical Attraction

**DOI:** 10.1155/2014/815690

**Published:** 2014-07-08

**Authors:** Abdon Atangana, P. D. Vermeulen

**Affiliations:** Institute for Groundwater Studies, Faculty of Natural and Agricultural Sciences, University of the Free State, Bloemfontein 9300, South Africa

## Abstract

We put into exercise a comparatively innovative analytical modus operandi, the homotopy decomposition method (HDM), for solving a system of nonlinear partial differential equations arising in an attractor one-dimensional Keller-Segel dynamics system. Numerical solutions are given and some properties show evidence of biologically practical reliance on the parameter values. The reliability of HDM and the reduction in computations give HDM a wider applicability.

## 1. Introduction

In 1970, Keller and Segel have offered parabolic systems to illustrate the aggregation process of cellular slime mold by the chemical attraction [1]. The system of a simplified form in the one-dimensional case is written as

(1) $$\begin{array}{c} \frac{\partial u\left(x,t\right)}{\partial t}=a\frac{\partial^{2}u\left(x,t\right)}{\partial x^{2}}-\frac{\partial }{\partial x}\left(u\left(x,t\right)\frac{\partial \chi \left(\rho \right)}{\partial x}\right),\\ \frac{\partial \rho \left(x,t\right)}{\partial t}=b\frac{\partial^{2}\rho \left(x,t\right)}{\partial x^{2}}+cu\left(x,t\right)-d\rho \left(x,t\right), \end{array}$$

subject to the boundaries conditions

(2) $$\begin{array}{c} \frac{\partial u\left(\alpha ,t\right)}{\partial x}=\frac{\partial u\left(\beta ,t\right)}{\partial x}=\frac{\partial \rho \left(\alpha ,t\right)}{\partial x}=\frac{\partial \rho \left(\beta ,t\right)}{\partial x}=0 \end{array}$$

and initial conditions

(3) $$\begin{array}{c} u\left(x,0\right)=u_{0}\left(x\right),\;\;\rho \left(x,0\right)=\rho_{0}\left(x\right),\;x\in I, \end{array}$$

where  *I* = (*α*, *β*) is a bounded open interval and *a*, *b*, *c*, and *d* are positive constants. The unknown functions *u*(*x*, *t*) and *ρ*(*x*, *t*) denote the concentration of amoebae and the concentration of chemical substance, respectively, in *I* × (0, *∞*). The chemotactic term (∂/∂*x*)(*u*(*x*, *t*)(∂*χ*(*ρ*)/∂*x*)) indicates that the cells are sensitive to the chemicals and are attracted by them. *χ*(*ρ*) called the sensitivity function is a smooth function of *ρ* ∈ (0, *∞*) which describes cell's perception and response to the chemical stimulus *ρ*. Several normalized forms have been suggested *ρ*, *ρ* ^2^, log⁡⁡(*ρ*), *ρ*/(*ρ* + 1) and *ρ* ^2^/(*ρ* ^2^ + 1), and so forth (see [2, 3]). Recently, the Keller-Segel (KS) equations attracted interests of many mathematicians. Since the modelling of chemotaxis has developed into a large and diverse discipline, one model which is widely used is the Keller-Segel model of chemotaxis; it is important to recall that chemotaxis describes the movement of single or multicellular organisms when they move up or down a chemical gradient [4]. This movement allows the organism to explore its extracellular environment. Organisms move randomly, away from repellents and towards attractants. Questions have arisen on how organisms can detect small changes in their extracellular environment [5]. Usually the organism will undergo a random walk, consisting of smooth swimming and brief direction changes (tumbles). By increasing the attractant, the tumbling is suppressed, which leads to a biased random walk [4]. The organism will then accumulate in areas of high attractant concentration. This type of movement is referred to as runs [5]. A combination of tumbles and runs allows the organism to explore and respond to changes in its extracellular environment [4].

The local solutions were studied by the second author [6]. It was also suggested in [6] that, in the one-dimensional case, (KS) possesses a global solution and that, in the two-dimensional case, when *χ*(*ρ*) = *kρ* (*k* being a positive constant) is a linear function, (KS) possesses a global solution for any sufficiently small initial function *u*_0_. Horstmann and Wang [7] showed more strongly that the global solution exists if the norm ||*u*_0_||_*L*^1^_ is smaller than a specific number, which is given from the coefficients of the equations. Recently, in the same case, the asymptotic behaviour of the global solutions was studied in [8]. On the other hand, Herrero and Velázquez [9] showed that when *χ*(*ρ*) is linear and the domain is a circular disc, there exist radial local solutions which blow up in a finite time. The blowup of nonradial local solutions was shown recently by [10, 11]. For the study of stationary solutions, we refer to [12–14]. In the field of dynamical systems theory some work has been carried out although the suggested models and algorithms are still in an introductory platform of establishing. It is perhaps important to notice that several analytical methods have been proposed to deal with nonlinear equations, but there exist a lot of nonlinear ordinary differential equations and nonlinear partial differential equations for which exact analytical solution cannot be found. There is no exact solution of (4) in the literature. To solve these problems, some eminent scholars have proposed some powerful iteration methods to deal with this class of nonlinear equation.

As V.M. Alexandrov wrote in the introduction of a well-liked science book* Asymtotology: Ideas, Methods, and Applications* [15, 16], asymptotic methods belong to the, perhaps, most romantic area of modern mathematics [15–19]. Though computer science is growing very fast and numerical simulation is applied everywhere, nonnumerical issues will still play a large role [16, 20–22]. There exist some alternative analytical asymptotic approaches such as the nonperturbative method, modified Lindstedt-Poincare method [21], variational iteration method [22], Adomian decomposition method [23], homotopy perturbation method [17, 24], and bookkeeping artificial parameter perturbation method [18].

The purpose of this paper is to derive analytical solutions of attractor one-dimensional Keller-Segel equations (1) via the relatively new analytical method the modified homotopy perturbation method. The HDM was recently used in [19, 25–27]. This method displays some advantages over existing methods.

The paper is prearranged as follows: in Section 2, we present the basic idea of the HDM for solving high orders differential equations. We present the application of the HDM for attractor one-dimensional Keller-Segel equations and numerical results in Section 3. In Section 4 we present the discussions. The conclusions are then given in Section 5.

## 2. Basic Properties of Homotopy Decomposition Method

With the purpose of making the fundamental possessions of the homotopy decomposition method [28] clear, we think about a universal nonlinear nonhomogeneous partial differential equation with initial conditions of the following form:

(4) ∂mU(x,t)∂tm=L(U(x,t))+N(U(x,t))+f(x,t),m=1,2,3….

Subject to the initial condition

(5) ∂iU(x,0)∂ti=yi(x),  ∂m−1U(x,0)∂tm−1=0,i=0,1,2…m−2,

*m* is the order of the derivative.

Where *f* is a known function, *N* is the general nonlinear differential operator and *L* represents a linear differential operator, and *m* is the order of the derivative. The method's first step is to apply the inverse operator of ∂^*m*^/∂*t* ^*m*^ on both sides of (4) to obtain

(6) U(x,t)=∑k=0m−1tkk!dku(x,0)dtk +∫0t∫0t1⋯∫0tm−1L(U(x,τ))+N(U(x,τ))        +f(x,τ)dτ⋯dt.

The multi-integral in (4) can be transformed to

(7) ∫0t∫0t1⋯∫0tm−1L(U(x,τ))+N(U(x,τ))+f(x,τ)dτ⋯dt =1(m−1)!∫0t(t−τ)m−1L(U(x,τ))        +N(U(x,τ))+f(x,τ)dτ,

so that (4) can be reformulated as

(8) U(x,t)=∑k=0m−1tkk!yi(x)+1(m−1)! ×∫0t(t−τ)m−1L(U(x,τ))+N(U(x,τ))   +f(x,τ)dτ.

Using the homotopy scheme the solution of the above integral equation is given in series form as

(9) $$\begin{array}{c} U\left(x,t,p\right)=\overset{\infty }{\underset{n=0}{\sum }}p^{n}U_{n}\left(x,t\right),\\ U\left(x,t\right)=\underset{p\to 1}{\lim}U\left(x,t,p\right) \end{array}$$

and the nonlinear term can be decomposed as

(10) $$\begin{array}{c} NU\left(r,t\right)=\overset{\infty }{\underset{n=1}{\sum }}p^{n}H_{n}\left(U\right), \end{array}$$

where *p* ∈ (0, 1] is an embedding parameter. *H*_*n*_(*U*) is the He's polynomials that can be generated by

(11) Hn(U0,…,Un)=1n!∂n∂pn[N(∑j=0npjUj(x,t))],n=0,1,2….

The modified homotopy perturbation method is obtained by the coupling of decomposition method with Abel integral and is given by

(12) ∑n=0∞pnUn(x,t) =T(x,t)+p1(m−1)!  ×∫0t(t−τ)m−1[f(x,τ)+L(∑n=0∞pnUn(x,τ))+∑n=0∞pnHn(U)]dτ

with

(13) $$\begin{array}{c} T\left(x,t\right)=\overset{m-1}{\underset{k=0}{\sum }}\frac{t^{k}}{k!}y_{i}\left(x\right). \end{array}$$

Comparing the terms of same powers of *p* produces solutions of various orders. The initial guess of the approximation is *T*(*x*, *t*) [26, 27]. This is actually the Taylor series of the exact solution of order *m*. Note that this initial guess insures the uniqueness of the series decompositions [26, 27].

## 3. Application

In this section we apply this method for solving coupled attractor one-dimensional Keller-Segel equations.

**Example 1 .**

Consider the following Keller Segel equation with the sensitivity function *χ*(*ρ*) = 1.

Then the chemotactic term

(14) $$\begin{array}{c} \frac{\partial }{\partial x}\left(u\left(x,t\right)\frac{\partial \chi \left(\rho \right)}{\partial x}\right)=0,\\ \frac{\partial u\left(x,t\right)}{\partial t}=a\frac{\partial^{2}u\left(x,t\right)}{\partial x^{2}},\\ \frac{\partial u\left(x,t\right)}{\partial t}=b\frac{\partial^{2}\rho \left(x,t\right)}{\partial x^{2}}+cu\left(x,t\right)-d\rho \left(x,t\right). \end{array}$$

Subject to the initial conditions

(15) $$\begin{array}{c} u\left(x,0\right)=me^{-x^{2}},\;\;\rho \left(x,0\right)=ne^{-x^{2}},\;x\in I. \end{array}$$

In the view of the HDM, we obtain the following equation:

(16)  ∑n=0∞pnun(x,t)−u(x,0)=p∫0ta∂2∂x2(∑n=0∞pnun(x,τ))dτ,∑n=0∞pnρn(x,t)+ρ(x,0) =p∫0tb∂2∂x2(∑n=0∞pnρn(x,τ))    +c∑n=0∞pnun(x,τ)−d∑n=0∞pnρn(x,τ)dτ.

Now comparing the terms of same power of *p*, we obtained the following integral equations:

(17) p0:u0(x,t)=u(x,0)=me−x2,  u0(x,0)=u(x,0),p0:ρ0(x,t)=ρ(x,0)=ne−x2,  ρ0(x,0)=ρ(x,0),p1:u1(x,t)=a∫0t∂2u0∂x2dτ,  u1(x,0)=0,p1:ρ1(x,t)=∫0tb∂2ρ0∂x2+cu0−dρ0dτ,  ρ1(x,0)=0,     ⋮pn:un(x,t)=a∫0t∂2un−1∂x2dτ,  un(x,0)=0,pn:ρn(x,t)=∫0tb∂2ρn−1∂x2+cun−1−dρn−1dτ,ρn(x,0)=0.

The following solutions are obtained straightforwardly:

(18) u(x,0)=me−x2,ne−x2,u1(x,t)=2ae−x2mt(−1+2x2),ρ1(x,t)=e−x2t(cm−n(d+b(2−4x2))),u2(x,t)=2a2e−x2mt2(3−12x2+4x4),ρ2(x,t) =12e−x2t2(d(−cm+nd)+2acm(−1+2x2)+2b(cm−2dn)(−1+2x2)+4b2n(3−12x2+4x4)),u3(x,t) =43a3e−x2mt3(−15+90x2−60x4+8x6)ρ3(x,t) =16e−x2t3(d2(cm−dn)+2bd(−2cm+3dn)×(−1+2x2)+4a2cm(3−12x2+4x2)+4b2(cm−3dn)(3−12x2+4x4)+8b3n(−15+90x2−60x4+8x6)+2acm(d−2dx2+b(6−24x2+8x4))).

Using the iterative formula, the remaining terms can be obtained. But here, only few terms of the series solutions are considered and the asymptotic solution is given as

(19) $$\begin{array}{c} u\left(x,t\right)=u_{0}\left(x,t\right)+u_{1}\left(x,t\right)+u_{2}\left(x,t\right)+u_{3}\left(x,t\right)+\cdots ,\\ \rho \left(x,t\right)=\rho_{0}\left(x,t\right)+\rho_{1}\left(x,t\right)+\rho_{2}\left(x,t\right)+\rho_{3}\left(x,t\right)+\cdots . \end{array}$$

The following figures show the biological behaviour of the coupled solutions for the following set of theoretical parameters: *m* = 120, *n* = 160, *a* = 0.5, *b* = 3, *c* = 1, and *d* = 2, first for a fixed time *t* = 5 and secondly for a fixed distance *x* = 1.

**Figure 1:**
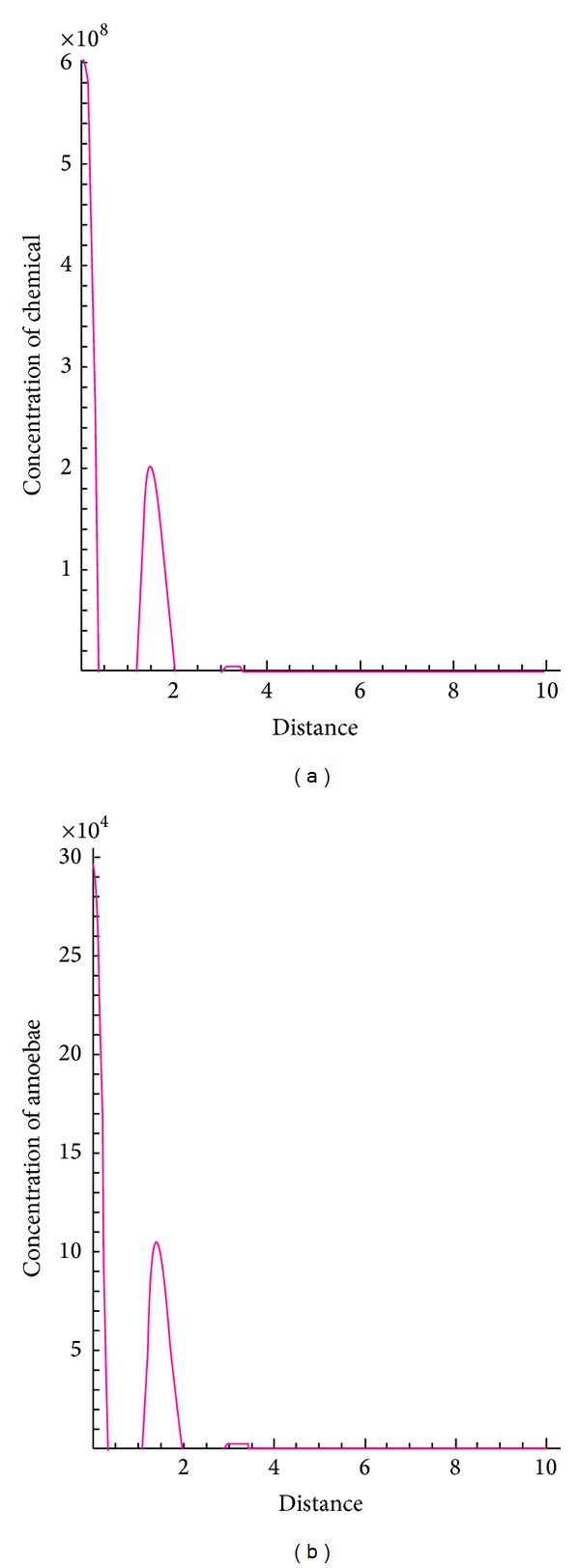
Biological behaviour of concentrations of the chemical substance and amoebae as function of space.

**Figure 2:**
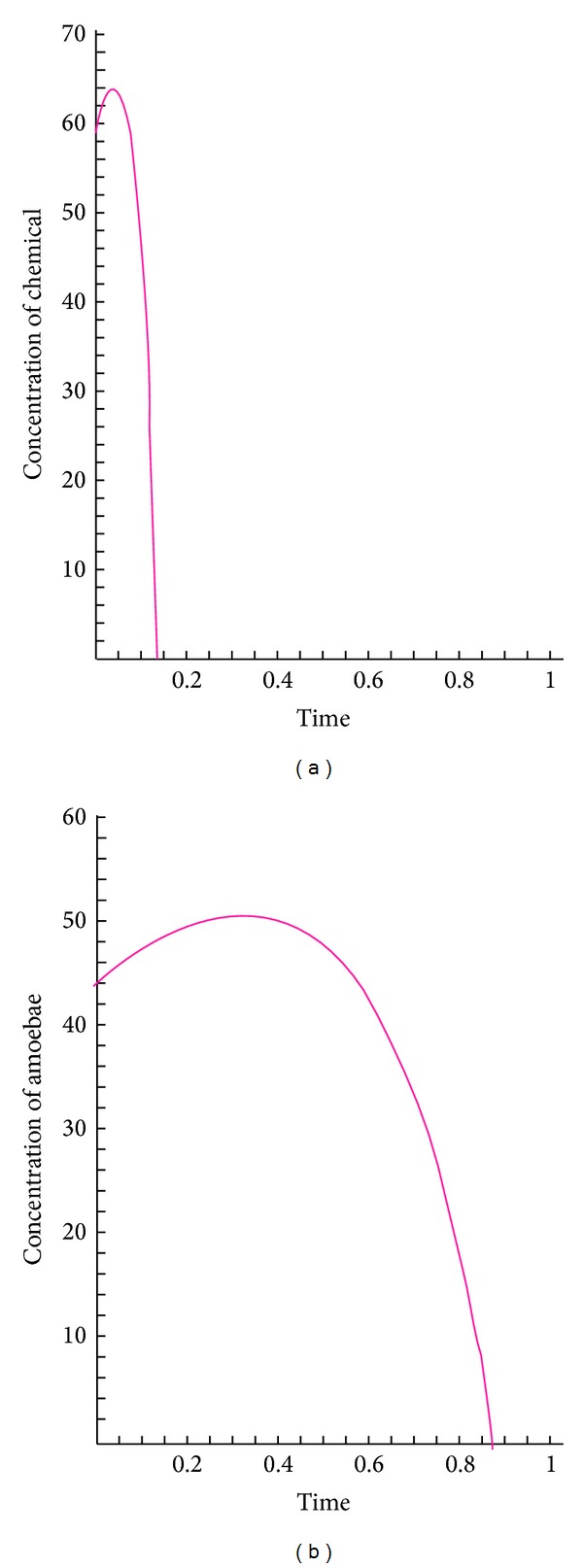
Biological behaviour of concentrations as function of time.

**Figure 3:**
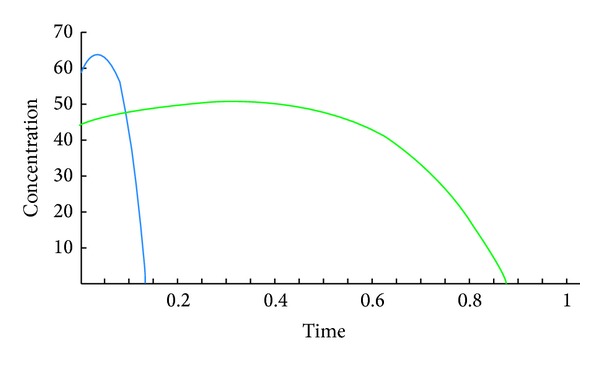
The behaviour of the coupled solutions.

Figures 1, 2, and 3 show the behaviour of the solution of the system of (15) describing the concentrations of the chemical substance and the amoebae in the human body. While on one hand, Figure 1 shows the behaviour as function of space, Figures 2 and 3 show the behaviour of these solutions as function of time. From the above figures, one can see that the concentration of amoebae reduces in space as the concentration of the chemical substance reduces. This simply implies that if the concentration of the chemical substance introduced in the human system to combat the spread of the disease is not sufficient enough, the amoebae will spread all over and the patient will certainly die. However, if this concentration is sufficient enough, the amoebae will decrease in space. It is observed from the graphical representation that the approximate solutions obtained here display the behaviour of the real world problem.

**Example 2 .**

Consider the following Keller-Segel equation with the sensitivity function *χ*(*ρ*) = *ρ*.

With the chemotactic term (∂/∂*x*)(*u*(*x*, *t*)(∂*χ*(*ρ*)/∂*x*)) = (∂*u*(*x*, *t*)/∂*x*)(∂*ρ*(*x*, *t*)/∂*x*) + *u*(*x*, *t*)(∂^2^ *ρ*(*x*)/∂*x* ^2^),

(20) $$\begin{array}{c} \frac{\partial u\left(x,t\right)}{\partial t}=a\frac{\partial^{2}u\left(x,t\right)}{\partial x^{2}}-\frac{\partial u\left(x,t\right)}{\partial x}\frac{\partial \rho \left(x,t\right)}{\partial x}+u\left(x,t\right)\frac{\partial^{2}\rho \left(x\right)}{{\partial x}^{2}},\\ \frac{\partial u\left(x,t\right)}{\partial t}=b\frac{\partial^{2}\rho \left(x,t\right)}{\partial x^{2}}+cu\left(x,t\right)+cu\left(x,t\right)-d\rho \left(x,t\right), \end{array}$$

subject to the initial conditions

(21) $$\begin{array}{c} u\left(x,0\right)=u_{0}\left(x\right),\;\;\rho \left(x,0\right)=\rho_{0}\left(x\right),\;x\in I. \end{array}$$

In the view of the homotopy decomposition method, we arrive at the following set of integral equations that are very easy to handle:

(22) p0:u0(x,t)=u0(x),  u0(x,0)=u(x,0),p0:ρ0(x,t)=ρ0(x),  ρ0(x,0)=ρ0(x),p1:u1(x,t)=∫0ta∂2u0∂x2−∂u0∂x∂ρ0∂x+u0∂2ρ0∂x2dτ,p1:ρ1(x,t)=∫0tb∂2ρ0∂x2+cu0−dρ0dτ,  ρ1(x,0)=0,      ⋮pn:un(x,t)=∫0t(a∂2un−1∂x2−∑j=0n−1∂uj∂x∂ρn−j−1∂x+∑j=0n−1uj∂2ρn−j−1∂x2)dτ,un(x,0)=0,pn:ρn(x,t)=∫0tb∂2ρn−1∂x2+cun−1−dρn−1dτ,ρn(x,0)=0.

Here we will consider two cases. Case one: we suppose that *u*(*x*, 0) = *me* ^−*x*^ and *ρ*(*x*, 0) = *ne* ^−*x*^. The following series solutions are obtained:

(23) u0(x,t)=me−x,  ρ0(x,t)=ne−x,u1(x,t)=ae−xmt,  ρ1(x,t)=e−x(cm+(b−d)n)t,u2(x,t)=12a2e−xmt2,ρ2(x,t)=12e−x(acm+(b−d)(cm+(b−d)n))t2,u3(x,t)=13!e−xm(at)3,ρ3(x,t) =13!e−xt3(a2cm+ac(b−d)m+(b−d)2(cm+(b−d)n)),u4(x,t)=14!e−xm(at)4,ρ4(x,t)=14!e−xt4(a3cm+a2c(b−d)m+ac(b−d)2m+(b−d)3(cm+(b−d)n))    ⋮

The remaining terms can be obtained by using the iteration formula. But here, only few terms of the series solutions are considered and the asymptotic solution is given as

(24) $$\begin{array}{c} u_{N}\left(x,t\right)=\overset{N}{\underset{n=0}{\sum }}me^{-x}\frac{{\left(at\right)}^{n}}{n!},\\ \rho \left(x,t\right)=\rho_{0}\left(x,t\right)+\rho_{1}\left(x,t\right)+\rho_{2}\left(x,t\right)+\rho_{3}\left(x,t\right)+\cdots . \end{array}$$

Therefore when *N* tends to infinity the concentration of amoebae converges to

(25) $$\begin{array}{c} u\left(x,t\right)=me^{\left(at-x\right)}. \end{array}$$

The following figures show the biological behaviour of the coupled solution for the following set of theoretical parameters: *m* = 120, *n* = 160, *a* = 0.5, *b* = 0.001, *c* = 1, and *d* = 2, first for a fixed time *t* = 5 and secondly for a fixed distance *x* = 5.

Second case, we suppose that

(26) $$\begin{array}{c} u\left(x,0\right)=me^{-x^{2}},\;\;\rho \left(x,0\right)=\;ne^{-x^{2}}. \end{array}$$

Following the homotopy decomposition steps, we arrived at the following series solutions:

(27) u(x,0)=me−x2,  ρ(x,0)=ne−x2,u1(x,t)=2e−2x2mt(−n+aex2(−1+2x2)),ρ1(x,t)=e−x2t(cm−n(d+b(2−4x2))),u2(x,t)=e−3x2mt2     ×(−cex2m−6aex2n(−1+2x2) +2a2e2x2(3−12x2+4x4) +n(dex2+2n+4nx2−6bex2(−1+2x2))),ρ2(x,t)=12e−2x2t2     ×(−cdex2m+d2ex2n−2cmn +2acex2m(−1+2x2) +2bex2(cm−2dn)(−1+2x2) +4b2ex2n(3−12x2+4x4)).

Using the iteration formulas, the remaining terms can be obtained. But here, only few terms of the series solutions are considered and the asymptotic solution is given as

(28) $$\begin{array}{c} u\left(x,t\right)=u_{0}\left(x,t\right)+u_{1}\left(x,t\right)+u_{2}\left(x,t\right)+u_{3}\left(x,t\right)+\cdots ,\\ \rho \left(x,t\right)=\rho_{0}\left(x,t\right)+\rho_{1}\left(x,t\right)+\rho_{2}\left(x,t\right)+\rho_{3}\left(x,t\right)+\cdots . \end{array}$$

The following figures show the biological behaviour of the coupled solutions for the following set of theoretical parameters: *m* = 120, *n* = 160, *a* = 0.5, *b* = 0.001, *c* = 1, and *d* = 2, first for a fixed time *t* = 5 and secondly for a fixed distance *x* = 5.

**Figure 4:**
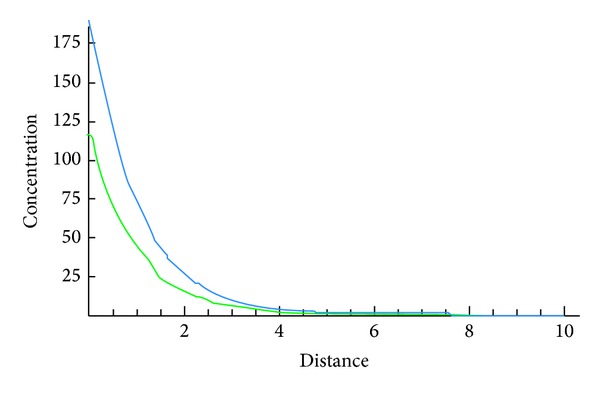
Coupled solutions.

**Figure 5:**
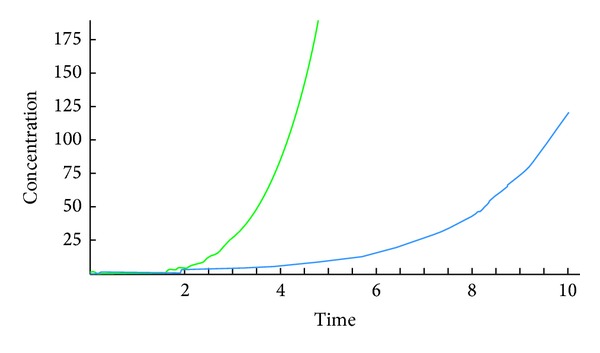
Coupled solutions.

**Figure 6:**
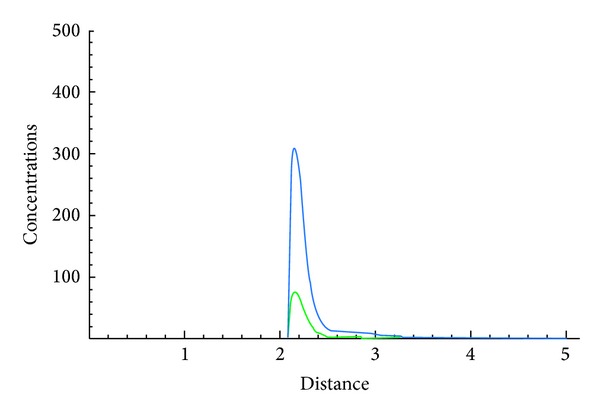
Coupled solutions.

**Figure 7:**
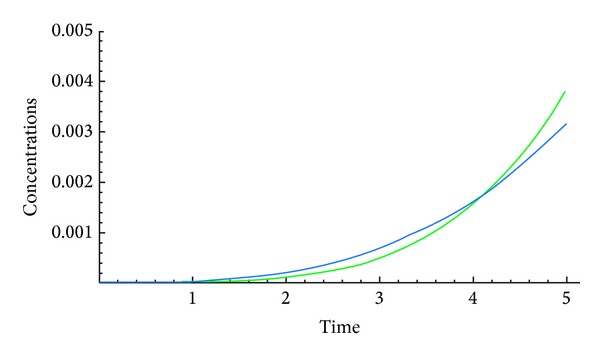
Coupled solutions.

The above figures show the behaviour of the solution of the system of (20) with initial conditions in (21) and (26). These solutions are describing the concentrations of the chemical substance and the amoebae in the human body for a given set of theoretical parameters chosen according to the literatures. While on one hand, Figure 4 shows the behaviour as function of space, Figures 5, 6, and 7 show the behaviour of these solutions as function of time. From the above figures, one can deduce that the concentration of amoebae reduces in space as the concentration of the chemical substance reduces. It is observed from the graphical representation that the approximate solutions obtained display the behaviour of the real world problem.

**Example 3 .**

Consider the following Keller-Segel equation with the sensitivity function *χ*(*ρ*) = *ρ* ^2^.

With the chemotactic term (∂/∂*x*)(*u*(*x*, *t*)(∂*χ*(*ρ*)/∂*x*)) = (∂*u*(*x*, *t*)/∂*x*)(∂*ρ* ^2^(*x*, *t*)/∂*x*) + *u*(*x*, *t*)(∂^2^ *ρ* ^2^(*x*, *t*)/∂*x* ^2^),

(29) ∂u(x,t)∂t =a∂2u(x,t)∂x2−∂u(x,t)∂x∂ρ2(x,t)∂x+u(x,t)∂2ρ2(x)∂x2,∂u(x,t)∂t=b∂2ρ(x,t)∂x2+cu(x,t)+cu(x,t)−dρ(x,t)

subject to the initial conditions

(30) $$\begin{array}{c} u\left(x,0\right)=u_{0}\left(x\right),\;\;\rho \left(x,0\right)=\rho_{0}\left(x\right),\;x\in I. \end{array}$$

Following the homotopy decomposition steps, we arrive at the following integral equations that are very easy to solve:

(31) p0:u0(x,t)=u0(x),  u0(x,0)=u(x,0),p0:ρ0(x,t)=ρ0(x),  ρ0(x,0)=ρ0(x),p1:u1(x,t)=∫0t(a∂2u0∂x2−2∂u0∂x∂ρ0∂xρ0+2u0ρ0∂2ρ0∂x2+2u0(∂ρ0∂x)2)dτ,p1:ρ1(x,t)=∫0tb∂2ρ0∂x2+cu0−dρ0dτ,ρ1(x,0)=0,pn:un(x,t) =∫0t(a∂2un−1∂x2−2∑j=0n−1∑k=0jρk∂uj−k∂x∂ρn−j−1∂x+2∑j=0n−1∑k=0jukρj−k∂2ρn−j−1∂x2+2∑j=0n−1∑k=0juk∂ρj−k∂x∂ρn−j−1∂x)dτ,un(x,0)=0,pn:ρn(x,t)=∫0tb∂2ρn−1∂x2+cun−1−dρn−1dτ,ρn(x,0)=0.

We will suppose that *u*_0_(*x*, 0) = *m*sin(*x*) and *ρ*(*x*, 0) = *n*sin(*x*). The following series solutions are obtained:

(32) u0(x,t)=msin(x),  ρ0(x,t)=nsin(x),u1(x,t)=−mt(a+2n2(cos⁡⁡(x))2)sin⁡(x),ρ1(x,t)=tsin(x)(cm−(b+d)n),u2(x,t) =−mtsin(x)(a+nt(2cm−an−2bn−2dn+n3+n3cos⁡⁡(2x))(sin⁡(x))2),ρ2(x,t) =−12t2(acm+bcm+cdm−b2n−2dbn−d2n+cnm2+cmn2cos⁡⁡(2x))sin⁡(x).

Using the iteration formulas, the remaining terms can be obtained. But here, only few terms of the series solutions are considered and the asymptotic solution is given as

(33) $$\begin{array}{c} u\left(x,t\right)=u_{0}\left(x,t\right)+u_{1}\left(x,t\right)+u_{2}\left(x,t\right)+u_{3}\left(x,t\right)+\cdots \\ \rho \left(x,t\right)=\rho_{0}\left(x,t\right)+\rho_{1}\left(x,t\right)+\rho_{2}\left(x,t\right)+\rho_{3}\left(x,t\right)+\cdots . \end{array}$$

## 4. Discussion

The homotopy decomposition method is chosen to solve this kind of nonlinear problem. Because of the following advantages that, the HDM has over the exiting methods. The method does not require the linearization or assumptions of weak nonlinearity [29, 30]. The solutions are not generated in the form of general solution as in Adomian decomposition method [29, 31, 32]. No Lagrange multiplier and correction functional are required as in the case of the variational iteration method [22, 30, 31, 34]. It is more realistic compared to the method of simplifying the physical problems. If the exact solution of the partial differential equation exists, the approximated solution via the method converges to the exact solution [26]. A construction of a homotopy *v*(*r*, *p*) : Ω × [0,1] is not needed as in the case of the homotopy perturbation method, because in this case one needs first to continuously deform a difficult problem into another one, which is easy to solve [17, 18, 24, 33]. HDM provides us with a convenient way to control the convergence of approximation series without adapting *h*, as in the case of [24] which is a fundamental qualitative difference in analysis between HDM and other methods [29–32, 34–38].

## 5. Conclusion

An interesting biological problem describing theaggregation process of cellular slime mold by the chemical attraction was investigated in this paper. We made use of the efficient method called homotopy decomposition method to derive the solution of the mathematical equation underpinning this problem. Analysis and results of nonlinear system of attractor one-dimensional Keller-Segel equation indicate that the model matches the regular biological diffusion behaviour observed in the field.
